# Complete genome sequences of 12 *Azospirillum* strains isolated from diverse agroecosystems

**DOI:** 10.1128/mra.00887-25

**Published:** 2026-04-20

**Authors:** Gabriele Bellotti, Marta Bisaschi, Edoardo Puglisi

**Affiliations:** 1 Department for Sustainable Food Process (DiSTAS), Università Cattolica del Sacro Cuore 96985 , Piacenza, Italy; University of Strathclyde, Glasgow, United Kingdom

**Keywords:** diazotrophs, biofertilizers, rhizobacteria, phytohormones, agroecosystems, genomics, plant-microbe interactions

## Abstract

We report complete genome sequences of 12 *Azospirillum* strains generated using a hybrid Oxford Nanopore–Illumina sequencing approach. High-quality assemblies reveal the full multi-replicon structure of each genome, providing a valuable resource for comparative genomics, taxonomy, and studies on environmental adaptation in plant-associated bacteria.

## ANNOUNCEMENT

*Azospirillum* are plant growth-promoting rhizobacteria known for their close associations with grasses and other crops. Several strains are commercially available due to their ability to fix atmospheric nitrogen and modulate phytohormones (1). In a previous work, some species were found to be misclassified, while many recognized species remain represented by a single genome (2). To address this gap, we acquired 10 *Azospirillum* strains from the Deutsche Sammlung von Mikroorganismen und Zellkulturen (DSMZ). In addition, two *A. argentinense* strains isolated in 2023 from tomato rhizospheres in Mali, cryopreserved in 20% glycerol at −20°C, were reactivated prior to sequencing to complete previous draft genomes (3).

DSMZ strains were obtained as freeze-dried cultures in sealed glass ampoules and revived following the supplier’s instructions. The Mali strains were activated from cryotubes on agar plates incubated aerobically at 30°C for 48 h. Colonies were transferred to DSMZ-221 broth and incubated for 24 h at 180 rpm. Cells were harvested, and genomic DNA was extracted using the EZNA Bacterial DNA Kit (Omega Bio-tek, USA), following the manufacturer’s protocol. DNA integrity number (DIN) was assessed using an Agilent 4500 TapeStation (High Sensitivity Kit; Agilent Technologies, USA), and purity was checked by NanoDrop (Thermo-Fisher Scientific, USA). Samples with DIN > 7, and A260/280 and A260/230 ratios >1.8 and 2.0, respectively, were sequenced.

ONT libraries were prepared using the Rapid Barcoding Kit 24 v14 (SQK-RBK114.24, ONT, UK) and loaded onto an R10.4.1 flow cell FLO-MIN114 on a MinION Mk1B device with MinKNOW v23.11.5. Raw signal data stored as POD5 were basecalled with Dorado v0.5.3 in super-accurate mode, generating FASTQ reads. Additional filtering was performed using NanoFilt v2.8.0, removing reads with Phred score <10 and reads <1,000 bp (4). From the same DNA extracts, Illumina short reads were generated by Novogene (Cambridge, UK) on a NovaSeq 6000 platform using PE250 technology. Library preparation followed the provider’s workflow with a custom kit, including DNA fragmentation, end repair, A-tailing, adapter ligation, size selection, and PCR amplification. Libraries were purified using the AMPure XP system (Beverly). The resulting libraries were assessed on an Agilent Fragment Analyzer system and quantified to 1.5 nM using Qubit fluorometry (Thermo-Fisher Scientific) and qPCR. According to the Novogene QC report, raw reads had average Phred scores between 30 and 40.

Unless otherwise noted, default parameters were used for all software. Both long-read and short-read sequencing data were used to generate assemblies. Long-read assemblies were generated using Flye v2.9.6 (5), Raven v1.8.3 (6), and Miniasm v03r179 (7). Trycycler v0.5.5 was used to compare assemblies, resolve structural differences, remove terminal overlaps, close gaps, and circularize replicons (8). The presence of multiple replicons was inferred from consistent recovery of multiple clusters, each yielding a distinct circular contig. Consensus assemblies were polished with Illumina short reads using Polypolish v0.6.0 (9), enabling the recovery of complete multi-replicon genomes typical of *Azospirillum*.

Genome completeness was assessed using BUSCO v6.0.0 (10). Assembly statistics are reported in Table 1. Genomes were annotated via BV-BRC v3.56.67 (11) and assigned taxID 191. The hybrid approach produced high-quality genomes suitable for downstream phylogenomic and taxonomic analyses.

**TABLE 1:** Read statistics, assembly quality metrics, and taxonomic assignment of the 12 *Azospirillum* genomes^*a*^

	Strain ID (DSMZ ID)	Ma4 (DSM 13400)	Sp 75 (DSM 2295)	Sp A2 (DSM 2297)	Sp A3a (DSM 1838)	Sp Br17 (DSM 1842)	Sp Col 2b (DSM 2293)	Sp Col 3 (DSM 2294)	Sp Col 5 (DSM 1860)	Sp RG20a (DSM 1840)	Sp USA 5b (DSM 2292)	UC4320	UC4321
Isolate data	Origin	Germany, Freising	Brazil	Nigeria, Ibadan	Senegal, Dakar	Brazil	Colombia, Papayan	Colombia, Papayan	Colombia, Papayan	Brazil	USA, Pullman	Mali, Bamako	Mali, Bamako
	Host/environment	*Miscanthus sinensis* roots	Maize roots	Maize roots	Grass roots	Maize roots	Maize roots	*Brachiaria* roots	*Hyparrhenia rufa* roots	Wheat roots	Agricultural soil	Tomato rhizosphere	Tomato rhizosphere
Genome data	Sample acc. no.	SAMEA120325263	SAMEA120325264	SAMEA120325265	SAMEA120325266	SAMEA120325267	SAMEA120325268	SAMEA120325269	SAMEA120325270	SAMEA120325271	SAMEA120325272	SAMEA120325273	SAMEA120325274
	Assembly acc. no.	GCA_977006905.2	GCA_977006955.2	GCA_977006985.2	GCA_977007015.2	GCA_977006935.2	GCA_977006915.2	GCA_977006925.2	GCA_977006975.2	GCA_977006945.2	GCA_977007025.2	GCA_977007005.2	GCA_977006995.2
	Genome coverage	76×	91×	113×	101×	55×	60×	50×	64×	70×	57×	82×	116×
Raw reads data	Illumina no. reads (×250 bp)	2,197,436	2,286,004	2,116,656	5,418,784	5,199,096	2,080,502	3,369,880	2,682,922	4,708,176	2,354,872	17,292,764	18,423,740
	Illumina reads acc. no.	ERR15701807	ERR15701810	ERR15701811	ERR15701813	ERR15701814	ERR15701815	ERR15701816	ERR15701838	ERR15701839	ERR15701840	ERR15701844	ERR15701845
	ONT tot reads length (bp)	27,313,058	544,419,509	475,034,816	216,270,867	183,833,901	307,558,085	368,846,639	34,798,674	57,623,640	113,354,585	39,818,961	629,231,055
	ONT no. reads	75,996.0	92,087.0	86,999.0	61,162.0	85,086.0	59,546.0	69,475.0	165,096.0	55,160.0	80,784.0	90,641.0	133,676.0
	ONT mean read length (bp)	3,962.4	4,010.5	4,141.9	5,223.0	4,474.6	2,825.5	3,306.1	1,635.4	4,755.1	4,690.4	4,311.8	3,374.7
	ONT reads acc. no.	ERR15700330	ERR15700317	ERR15700344	ERR15700345	ERR15700347	ERR15700386	ERR15700398	ERR15700400	ERR15700404	ERR15700432	ERR15700433	ERR15701805
BUSCO	Completeness (%)	100	100	100	100	100	100	99.8	100	100	100	100	100
	Fragmented BUSCOs (%)	0.0	0.0	0.0	0.0	0.0	0.0	0.0	0.0	0.0	0.0	0.0	0.0
	Missing BUSCOs (%)	0.0	0.0	0.0	0.0	0.0	0.0	0.0	0.0	0.0	0.0	0.0	0.0
QUAST	No. of contigs	7	6	7	8	8	8	7	8	8	7	8	7
	Total genome size (bp)	7,501,717	6,863,639	7,591,753	7,106,214	7,379,528	7,719,855	6,977,525	8,855,533	7,380,181	6,770,114	7,470,993	7,172,418
	GC content (%)	68.8	69.1	68.4	68.3	67.8	67.7	67.7	67.5	67.8	67.8	68.6	69.0
	N50 (bp)	1,357,979	1,822,607	2,064,099	1,107,683	1,154,421	1,085,136	1,348,664	1,192,050	1,154,421	976,033	1,960,025	1,788,884
Taxonomy	DSMZ reported as	*Azospirillum doebereinerae*	*Azospirillum brasilense*	*Azospirillum brasilense*	*Azospirillum largimobile*	*Azospirillum* sp.	*Azospirillum largimobile*	*Azospirillum largimobile*	*Azospirillum largimobile*	*Azospirillum largimobile*	*Azospirillum largimobile*	–^*b*^	–
	Closest type strain	*Azospirillum doebereinerae* GSF71^T^ GCA_003989665.1	*Azospirillum argentinense* Az39^T^ GCA_000632475.2	*Azospirillum argentinense* Az39^T^ GCA_000632475.2	*Azospirillum humicireducens* SgZ-5^T^ GCA_001639105.2	*Azospirillum palustre* B2^T^ GCA_002573965.1	*Azospirillum palustre* B2^T^ GCA_002573965.1	*Azospirillum melinis* TMCY0552^T^ GCA_013340935.1	*Azospirillum lipoferum* Sp 59b^T^ GCA_008364955.1	*Azospirillum palustre* B2^T^ GCA_002573965.1	*Azospirillum ramasamyi* M2T2B2^T^ GCA_003233655.1	*Azospirillum argentinense* Az39^T^ GCA_000632475.2	*Azospirillum argentinense* Az39^T^ GCA_000632475.2
	ANI (%)	93.63	94.81	98.35	92.60	96.23	95.96	96.60	90.93	96.23	93.21	98.40	95.20

^*a*^ Read statistics include ENA accession numbers, Illumina and Oxford Nanopore read counts, mean ONT read length, and long-read coverage. Assembly quality metrics were calculated using QUAST v5.3.0 (12). Genome completeness was assessed using BUSCO v6.0.0 (10) with the marker set rhodospirillales_odb10. Taxonomic assignments were based on ANI comparisons against type strain genomes using FastANI v1.1 (13).

^*b*^ "–", Strains not in DSMZ collection.

## Data Availability

This Whole Genome Shotgun project, named “Complete genome sequences of *Azospirillum* strains,” has been deposited in the European Nucleotide Archive (ENA) under the project accession PRJEB100579. Raw reads and assemblies are available under the accession numbers reported in Table 1.

## References

[1] Cassán F, Coniglio A, López G, Molina R, Nievas S, de Carlan CLN, Donadio F, Torres D, Rosas S, Pedrosa FO, de Souza E, Zorita MD, de-Bashan L, Mora V. 2020. Everything you must know about Azospirillum and its impact on agriculture and beyond. Biol Fertil Soils 56:461–479. doi:10.1007/s00374-020-01463-y

[2] Bellotti G, Cortimiglia C, Antinori ME, Cocconcelli PS, Puglisi E. 2025. Comprehensive genome-wide analysis for the safety assessment of microbial biostimulants in agricultural applications. Microb Genom 11:001391. doi:10.1099/mgen.0.001391 40294085 PMC12038027

[3] Bellotti G, Sanou P, Sunzini P, Tabaglio V, Cocconcelli PS, Puglisi E. 2024. Draft genome sequences of four plant-associated free-living diazotrophs isolated from the rhizosphere of Malian tomato. Microbiol Resour Announc 13:e0056824. doi:10.1128/mra.00568-24 39436066 PMC11555994

[4] De Coster W, D’Hert S, Schultz DT, Cruts M, Van Broeckhoven C. 2018. NanoPack: visualizing and processing long-read sequencing data. Bioinformatics 34:2666–2669. doi:10.1093/bioinformatics/bty149 29547981 PMC6061794

[5] Lin Y, Yuan J, Kolmogorov M, Shen MW, Chaisson M, Pevzner PA. 2016. Assembly of long error-prone reads using de Bruijn graphs. Proc Natl Acad Sci USA 113:E8396–E8405. doi:10.1073/pnas.1604560113 27956617 PMC5206522

[6] Vaser R, Šikić M. 2021. Time- and memory-efficient genome assembly with Raven. Nat Comput Sci 1:332–336. doi:10.1038/s43588-021-00073-4 38217213

[7] Li H. 2016. Minimap and miniasm: fast mapping and de novo assembly for noisy long sequences. Bioinformatics 32:2103–2110. doi:10.1093/bioinformatics/btw152 27153593 PMC4937194

[8] Wick RR, Judd LM, Cerdeira LT, Hawkey J, Méric G, Vezina B, Wyres KL, Holt KE. 2021. Trycycler: consensus long-read assemblies for bacterial genomes. Genome Biol 22:266. doi:10.1186/s13059-021-02483-z 34521459 PMC8442456

[9] Bouras G, Judd LM, Edwards RA, Vreugde S, Stinear TP, Wick RR. 2024. How low can you go? Short-read polishing of Oxford Nanopore bacterial genome assemblies. Microb Genom 10:001254. doi:10.1099/mgen.0.001254 38833287 PMC11261834

[10] Simão FA, Waterhouse RM, Ioannidis P, Kriventseva EV, Zdobnov EM. 2015. BUSCO: assessing genome assembly and annotation completeness with single-copy orthologs. Bioinformatics 31:3210–3212. doi:10.1093/bioinformatics/btv351 26059717

[11] Wattam AR, Abraham D, Dalay O, Disz TL, Driscoll T, Gabbard JL, Gillespie JJ, Gough R, Hix D, Kenyon R, et al.. 2014. PATRIC, the bacterial bioinformatics database and analysis resource. Nucleic Acids Res 42:D581–D691. doi:10.1093/nar/gkt1099 24225323 PMC3965095

[12] Gurevich A, Saveliev V, Vyahhi N, Tesler G. 2013. QUAST: quality assessment tool for genome assemblies. Bioinformatics 29:1072–1075. doi:10.1093/bioinformatics/btt086 23422339 PMC3624806

[13] Jain C, Rodriguez-R LM, Phillippy AM, Konstantinidis KT, Aluru S. 2018. High throughput ANI analysis of 90K prokaryotic genomes reveals clear species boundaries. Nat Commun 9:5114. doi:10.1038/s41467-018-07641-9 30504855 PMC6269478

